# Ultrasonic-Assisted Incremental Microforming of Thin Shell Pyramids of Metallic Foil

**DOI:** 10.3390/mi8050142

**Published:** 2017-05-03

**Authors:** Toshiyuki Obikawa, Mamoru Hayashi

**Affiliations:** Institute of Industrial Science, The University of Tokyo, Tokyo 153-8505, Japan; mamolu@ba2.so-net.ne.jp

**Keywords:** incremental microforming, ultrasonic spindle, shin shell micropyramid, metallic foil, forming limit, shape accuracy

## Abstract

Single point incremental forming is used for rapid prototyping of sheet metal parts. This forming technology was applied to the fabrication of thin shell micropyramids of aluminum, stainless steel, and titanium foils. A single point tool used had a tip radius of 0.1 mm or 0.01 mm. An ultrasonic spindle with axial vibration was implemented for improving the shape accuracy of micropyramids formed on 5–12 micrometers-thick aluminum, stainless steel, and titanium foils. The formability was also investigated by comparing the forming limits of micropyramids of aluminum foil formed with and without ultrasonic vibration. The shapes of pyramids incrementally formed were truncated pyramids, twisted pyramids, stepwise pyramids, and star pyramids about 1 mm in size. A much smaller truncated pyramid was formed only for titanium foil for qualitative investigation of the size reduction on forming accuracy. It was found that the ultrasonic vibration improved the shape accuracy of the formed pyramids. In addition, laser heating increased the forming limit of aluminum foil and it is more effective when both the ultrasonic vibration and laser heating are applied.

## 1. Introduction

Functional miniaturized structures of various materials will be widely applied to sensors, filters, biotesters, bioscaffold, etc. in the near future. For this purpose, efficient microprocessing technologies with high accuracy and high surface quality are required to meet the demand for their production. Microcutting [1], micro-laser-machining [2], micro-electric discharge machining [3,4], and microforming [5] have been intensively studied, and recently, micro-additive-manufacturing combined with other microprocessing [6] is more and more important in tailor-made manufacturing or high-mix low-volume production of complex parts.

Incremental forming with a single point tool is regarded as a rapid prototyping process for sheet metal forming because no die is needed and parts of free-form surfaces can be fabricated incrementally [7]. The forming limit is much larger for this technology than for other forming methods [8]. Therefore, this technology can be applied to the fabrication of various shapes of sheet metal parts [9]. It also can be applied to microforming of the miniature shell structures of aluminium, gold, and stainless steel foils [10,11,12,13,14]. The preparation of backing plates of various shapes, which are used as a support in ordinary incremental forming, is not easy for incremental forming of microparts. For this reason, no backing plate is needed for the developed microforming technologies. However, the accuracy of microshell structures formed without using a backing plate is not so high, and thus, it should be improved without reducing the forming limit.

In this paper, ultrasonic assisted incremental microforming technology has been developed using an ultrasonic spindle for improving the accuracy of microshell structures of aluminium, stainless steel, and titanium foils. Laser heating was also applied to the ultrasonic assisted incremental microforming for softening and increasing formability of aluminum foil under deformation. The forming limit and forming accuracy of micropyramids were investigated under the conditions of with and without ultrasonic vibration and laser heating. Only for a foil of titanium—a difficult-to-work material—a micropyramid in a size of 283 μm was also formed under the conditions of ultrasonic vibration for qualitatively investigating the size reduction on forming accuracy.

## 2. Materials and Methods

**Figure 1:**
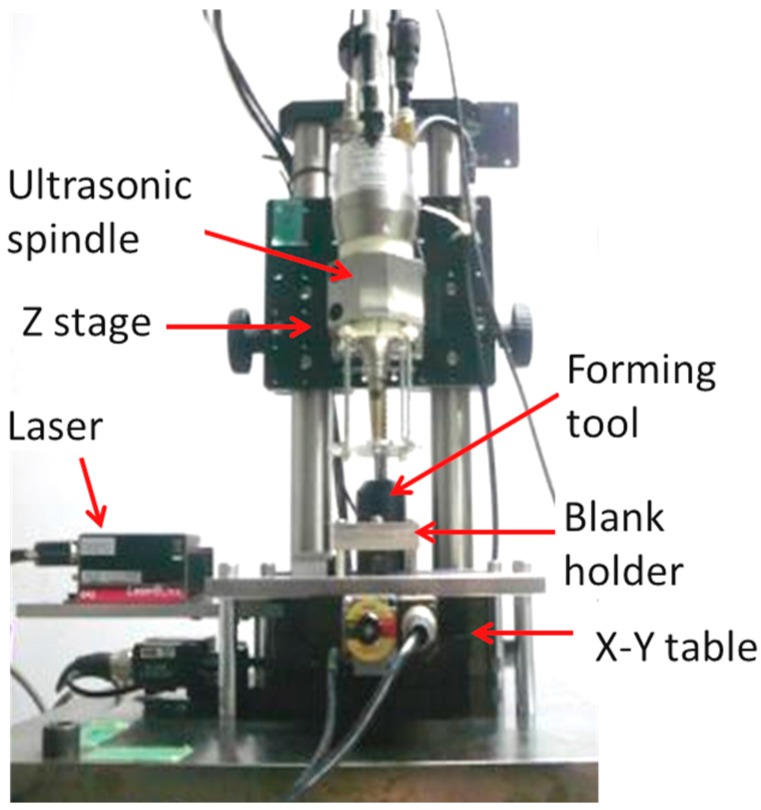
Desktop microforming machine.

An incremental microforming machine with an ultrasonic spindle is shown in Figure 1. It is placed on a vibration-free table for insulating external disturbances. It is composed of an x-y table, z stage, ultrasonic spindle with a Langevin ultrasonic transducer, forming tool, blank holder, base, column, and laser source and its optical system. An ordinary motor spindle used in the original and improved forming machines [10,11,12,13] has been replaced with an ultrasonic spindle and laser source and its optical system has been newly installed. The ultrasonic spindle causes vibration in the axial direction of the spindle. The vibration frequency is 42.5 kHz and its amplitude is set to be 0.5 μm, the minimum amplitude of the spindle. The x-y table and z-stage are controlled with a personal computer numerically. Their resolutions of motion are 0.01 μm, which is small enough to fabricate a shell structure of the order of one millimeter.

**Figure 2:**
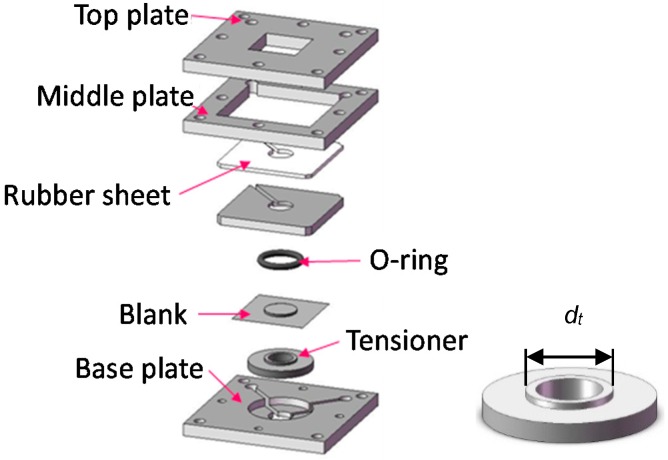
Blank holder and tensioner.

Aluminum foil of type 8021 used for the experiments is 6.5 and 12 μm-thick, while stainless steel foil of type 304 is 8 μm-thick and titanium foil is 5 μm-thick. The 8021 foil has a chemical composition listed in Table 1 and a crystal grain size of 5–10 μm [15]. The elongation of metallic foils decreases with decreasing thickness. It is about five percent for the aluminum foils [15] and about one percent for stainless foil [16]. A blank sheet is put on matte side up between the tensioner and O-ring, and then, it was clamped in a blank holder as shown in Figure 2. The holder can apply an appropriate size of tension to a blank by adjusting the sizes of a tensioner and O-ring [10,11,12]. This method for applying tension to a blank was also adopted in reference [14]. As described elsewhere [10,11,12] in details, no backing plate supporting a blank is used because miniaturization of a backing plate is not easy.

**Figure 3:**
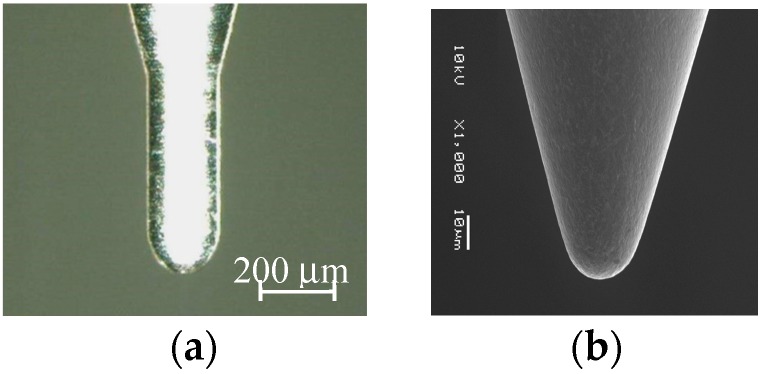
Microtool for incremental microforming: (**a**) *R* = 100 μm; (**b**) *R* = 10 μm.

A single point forming tool of ultra-fine grain cemented carbide with a tip radius of *R* = 100 μm or 10 μm is used in this study (Figure 3). Before starting microforming, z-position of the top surface of a blank was determined accurately by detecting the contact between the tool and blank. n-propyl alcohol is used as a lubricant for avoiding adhesion and abrasion between the forming tool and blank. High speed rotation of the tool generates the hydrodynamic pressure between the rotating tool and blank being formed. Under this condition, the alcohol may penetrate into the interface between them if the contact stress is low.

**Figure 4:**
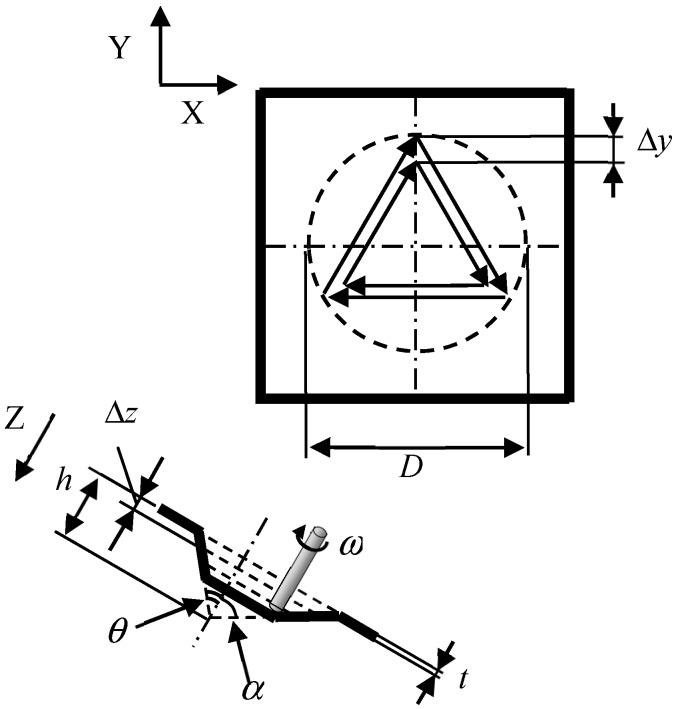
Schematic diagram of tool path and foil deformation during forming of a triangular pyramid.

Incremental microforming process of a triangular pyramid, for example, is shown in Figure 4, where *D* is the diameter of a circumscribed circle of a base of a pyramid, *α* is a half apex angle defined as an angle between a lateral edge and a vertical line through an apex, *θ* is a half apex angle defined as an angle between a triangular lateral face and the vertical line, *t* is the thickness of a blank, *ω* is the tool rotational speed, and Δ*z* is the axial feed per a planar tool path. The side length of a triangular tool path on a plane shrinks step by step. The tool rotational speed *ω* and table speed *v_t_* were set to be 5000 min^−1^ and 200 μm/s, respectively, except that *ω* = 10,000 min^−1^ and *v_t_* = 150 μm/s for a titanium micropyramid of *D* = 283 μm. The axial feed Δ*z* was 5 and 12 μm for 6.5 and 12 μm-thick aluminum foils, respectively, 5 μm for 8 μm-thick stainless steel foil, and 2 and 1 μm for titanium pyramids of *D* = 1.41 mm and *D* = 283 μm, respectively. When the forming was conducted under the condition of laser heating, the forming area of the foil was irradiated from the top of a pyramid (from the below of a pyramid) using a semiconductor laser of 130 mW; the laser beam was reflected using a prism under the blank holder.

**Figure 5:**
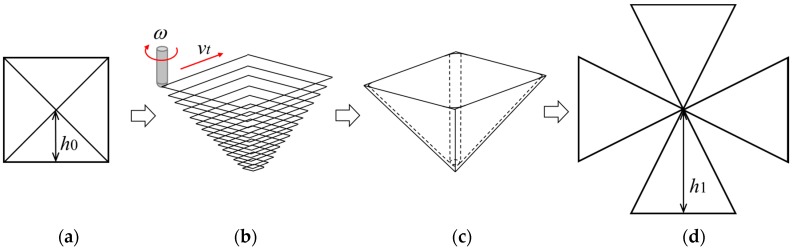
Forming of a square pyramid: (**a**) square blank to be formed; (**b**) tool path; (**c**) formed pyramid; (**d**) development view of a square pyramid.

The triangular lateral faces of a square pyramid elongates from *h*_0_ to *h*_1_ by single point incremental forming as shown in Figure 5. Its elongation *e* in percent is given by

*e* = 100(*h*_1_/*h*_0_ − 1) = 100(cosec *θ* − 1) (1)

while the logarithmic strain *ε* corresponding to the elongation *e* is

*ε* = ln(*h*_1_/*h*_0_) = ln(cosec *θ*) (2)

Both the elongation and strain increase with decreasing half apex angle. Equations (1) and (2) are applied for truncated pyramids with the same half apex angle *θ*.

## 3. Results and Discussion

**Figure 6:**
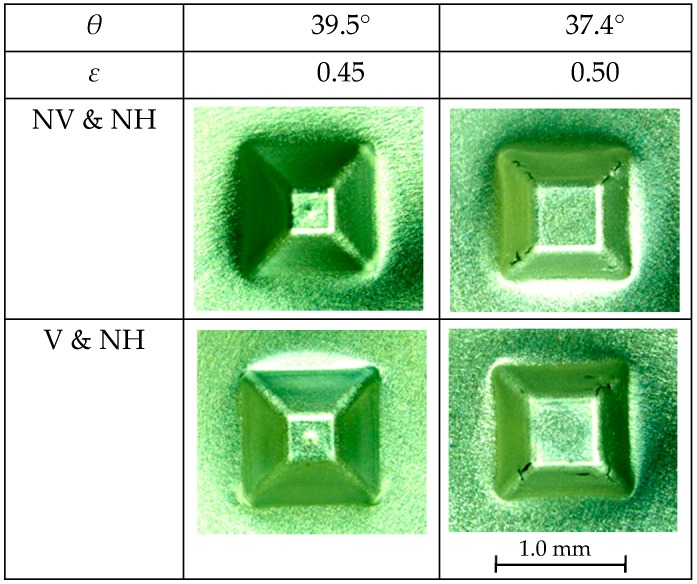
Forming limits for truncated pyramids with and without ultrasonic vibration (V and NV) and without laser heating (NH).

**Figure 7:**
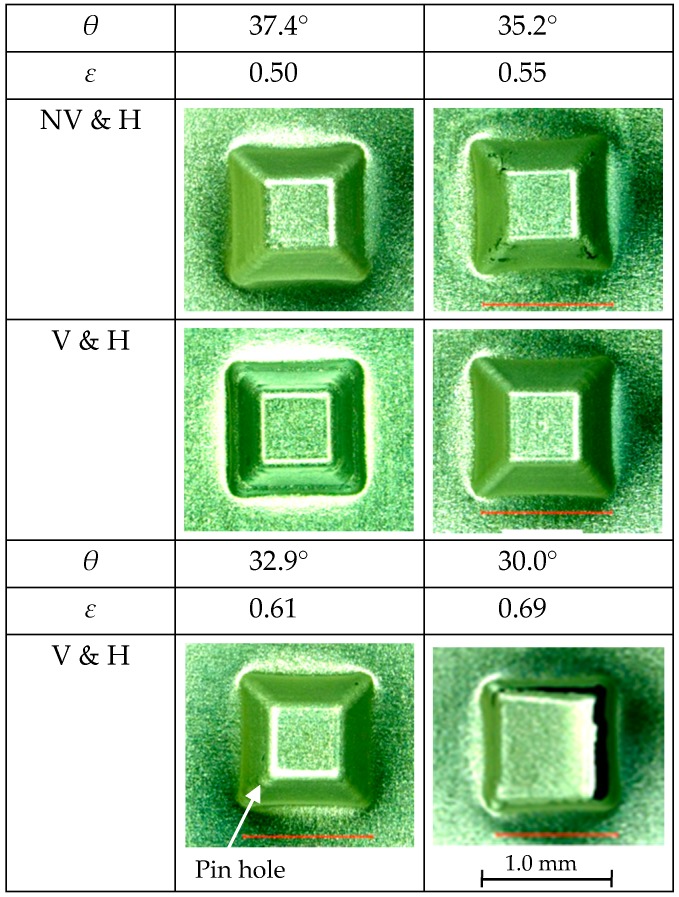
Forming limits for truncated pyramids with and without ultrasonic vibration (V and NV) and with laser heating (H).

The influence of ultrasonic vibration on the forming limit of a truncated pyramid of 6.5 μm-thick aluminum foil is shown in Figure 6, whereas, those of ultrasonic vibration and laser heating on the forming limit is shown in Figure 7. The diameter of a circumscribed circle of a square pyramid base *D* was 1.41 mm. Forming conditions of with and without ultrasonic vibration are denoted as V and NV, respectively and those of with and without laser heating are denoted as H and NH, respectively. In these figures, the values of half apex angle *θ* and corresponding strain *ε* are written in rows above micrographs of the top views of formed pyramids. The value of *θ* is changed step by step so that the value of *ε* increases by about 0.05. 

It is seen in Figure 6 that square pyramids of *θ* = 39.5° were formed without cracks under the conditions of with and without ultrasonic vibration, but cracks grew especially along the lateral edges of pyramids of *θ* = 37.4° formed under both the conditions. Thus, the forming limit *ε*_c_ was 0.45 for the condition of without laser heating and the ultrasonic vibration increased the forming limit marginally. The fact that the forming limit obtained above is less than those obtained in the previous research works [11,12] is partly because n-propyl alcohol is a poor lubricant compared with pure water used in the previous research and partly because the thickness of aluminium foil was reduced from 12 μm to 6.5 μm.

In the case of laser heating, it is found from Figure 7 that *ε*_c_ was 0.55 and 0.50 for the conditions of with and without ultrasonic vibration, respectively. This indicates that the laser heating can increase the forming limit of the aluminum foil and it is more effective when the ultrasonic vibration is applied. Because only a pinhole was found on a pyramid of *θ* = 32.9° under the conditions of with both ultrasonic vibration and laser heating, optimization of forming parameters may increase the forming limit under these conditions. It should be noted that elongation for *θ* = 35.2°, which is calculated to be 73.4% from Equation (1), is more than 10 times as large as that of about 5% obtained for tensile test. 

In addition to the formability, it is confirmed that almost all the cracks appeared on the lateral edges of formed pyramids. This fact is consistent with the results obtained in reference [13], that a crack nucleated on a pyramid edge in the microforming of aluminum foil, whereas it nucleated on a triangular pyramid face in the microforming of stainless steel foil. 

**Figure 8:**
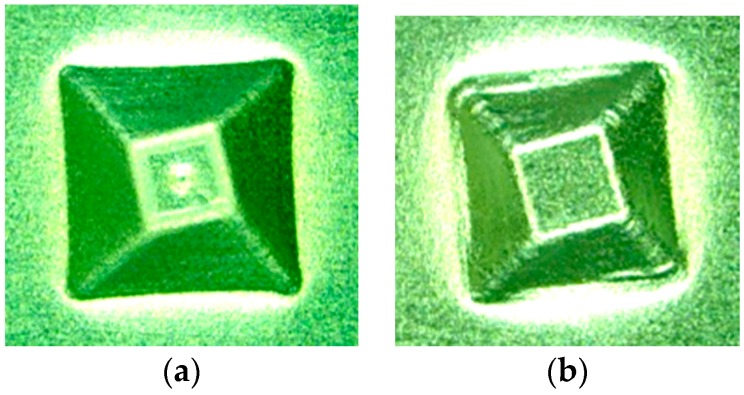
Top views of twisted pyramids formed: (**a**) with ultrasonic vibration; (**b**) without ultrasonic vibration.

**Figure 9:**
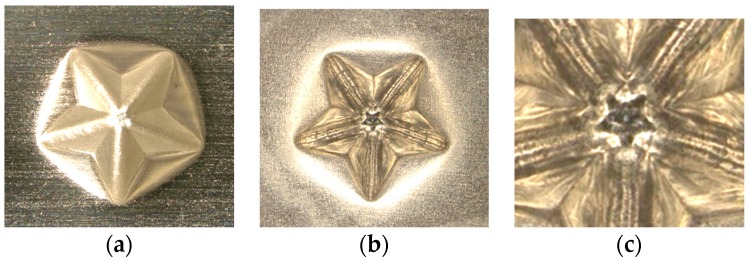
Top and bottom views of a star pyramid formed with ultrasonic vibration: (**a**) top view; (**b**) bottom view; (**c**) magnified center part of bottom view.

**Figure 10:**
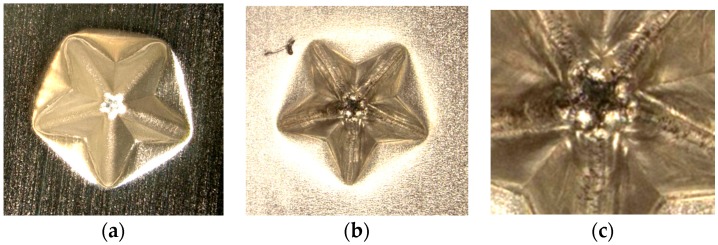
Top and bottom views of a star pyramid formed without ultrasonic vibration: (**a**) top view; (**b**) bottom view; (**c**) magnified center part of bottom view.

**Figure 11:**
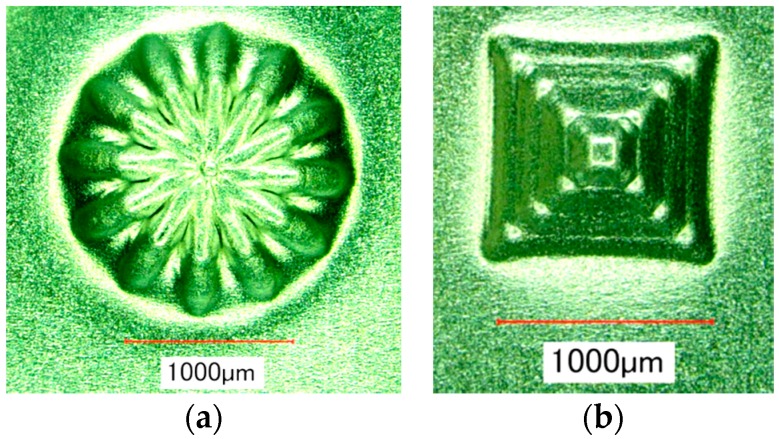
Other pyramids formed by ultrasonic incremental microforming: (**a**) pyramid like sunflower; (**b**) stepwise pyramid.

The effect of ultrasonic vibration assistance on the shape accuracy in incremental microforming was investigated by forming a twisted pyramid of 6.5 μm-thick aluminum foil and a star pyramid of 12 μm-thick aluminum foil with and without ultrasonic vibration. Diameter *D* was 1.41 mm for the twisted pyramid and 1.60 mm for the star pyramid. Figure 8 shows the top views of the twisted pyramids incrementally formed. The surfaces and edges of the pyramid made by ultrasonic assisted microforming are much smoother and not wavier than those by ordinary microforming without ultrasonic vibration. The top and bottom views of star pyramids formed with and without ultrasonic vibration are shown in Figure 9 and Figure 10, respectively. It is seen that not only the convex parts but also the concave parts are formed well, much better than expected. According to the top views, there does not seem to be a significant difference in the shape accuracy between the two pyramids formed with and without ultrasonic vibration. However, it is confirmed from the bottom views and more clearly from the magnified center of the bottom views that the traces of the tool path near the center of pyramid on the bottom surface is a nearly regular pentagon for ultrasonic assisted microforming, whilst it is heavily distorted for the ordinary microforming. Results in Figure 8, Figure 9 and Figure 10 prove that the ultrasonic vibration can improve the shape accuracy of the formed pyramids. Other pyramids formed in this study using aluminum foil are a pyramid-like sunflower of *D* = 1.72 mm and a stepwise pyramid of *D* = 1.41 mm shown in Figure 11. They were formed well using ultrasonic incremental microforming.

**Figure 12:**
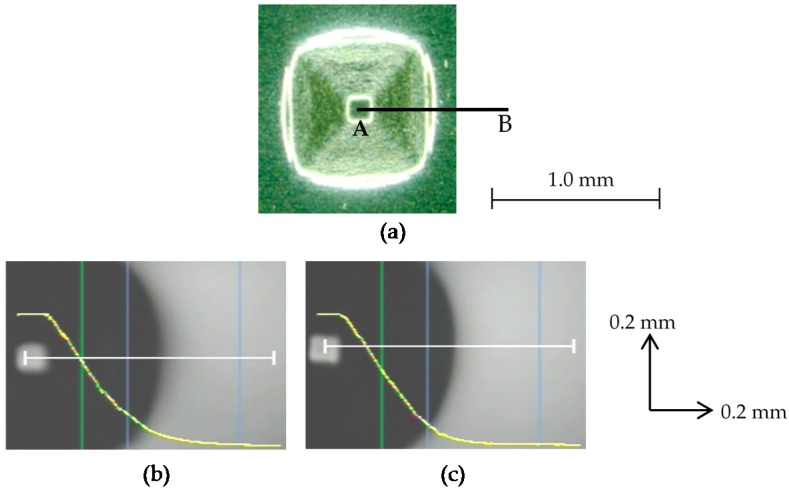
Profiles of pyramids of stainless steel foil under different conditions: (**a**) top view of a pyramid formed without ultrasonic vibration and heating; (**b**) surface profile along AB in the case of NV and NH; (**c**) surface profile along AB in the case of V and H.

As described above, the ultrasonic vibration was effective in improving the shape accuracy of rather complicated pyramids such as a twisted pyramid and a star pyramid, whilst the ultrasonic vibration and laser heating were not so effective for truncated pyramids. In contrast, they were able to improve the shape accuracy of a truncated pyramid of 8 μm-thick stainless steel foil, which is much stiffer than 6.5 μm-thick aluminum foil. Two pyramids of stainless steel foil of *D* = 1.0 mm and *θ* = 45° were formed under NV and NH conditions and under V and H conditions. Their profiles measured with a confocal laser displacement meter along line AB of an attached figure are shown in Figure 12b,c. It is seen that the line of lateral face is connected with the base line via a curved line with a small radius under V and H conditions. 

**Figure 13:**
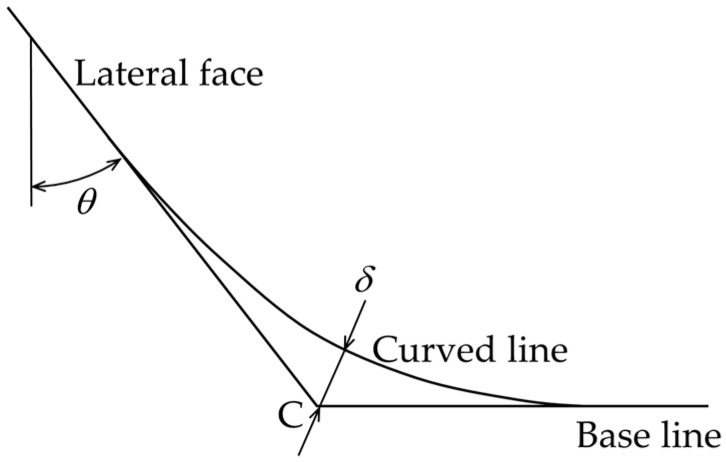
The forming accuracy based on distance *δ* from point C to the pyramid profile.

The forming accuracy was evaluated based on distance *δ* from point C to the pyramid profile as shown in Figure 13. The value of *δ* was 43 μm and 26 μm for NV and NH conditions and V and H conditions, respectively. The theoretical value of the distance *δ_th_* for perfect forming is given by

(3) $$\delta_{th}=R\left[\sqrt{1+\tan^{2}\left(\frac{\pi -2\theta }{4}\right)}-1\right]$$

and hence, the forming error can be defined by *δ* − *δ_th_*. Because the value of *δ_th_* is calculated to be 8.2 μm for *R* = 100 μm and *θ* = 45°, the ultrasonic vibration and laser heating reduced the forming error by half. However, neither the ultrasonic vibration nor the laser heating increased the forming limit of a square pyramid of stainless steel foil: *ε*_c_ was 0.38 for (NV & NH) and (V & NH); it was slightly reduced to 0.35 for (NV & H) and (V & H). This is probably because it is difficult to heat the forming area uniformly due to very low heat conductivity of stainless steel.

**Figure 14:**
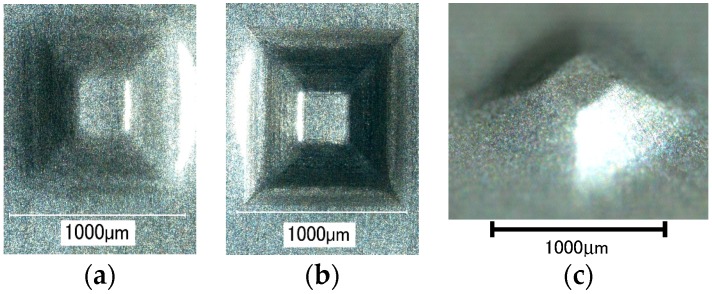
Titanium pyramids of *D* = 1.41 mm formed with ultrasonic vibration: (**a**) top view; (**b**) bottom view; (**c**) high angle view.

**Figure 15:**
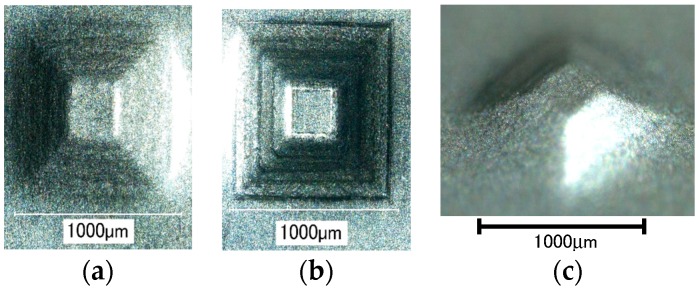
Titanium pyramids of *D* = 1.41 mm formed without ultrasonic vibration: (**a**) top view; (**b**) bottom view; (**c**) high angle view.

Thin shell pyramids of titanium foil of *D* = 1.41 mm and *θ* = 50° formed with and without ultrasonic vibration are shown in Figure 14 and Figure 15, respectively. It was seen that wrinkles were caused on the lateral faces by incremental microforming when ultrasonic vibration was not applied. In contrast, they almost disappeared and feed marks of a single point tool were seen only on the back surface of a pyramid when ultrasonic vibration was applied. It should be noted that the ultrasonic vibration effectively removed the wrinkles in a difficult forming process of the twisted pyramid in Figure 8 as described above.

**Figure 16:**
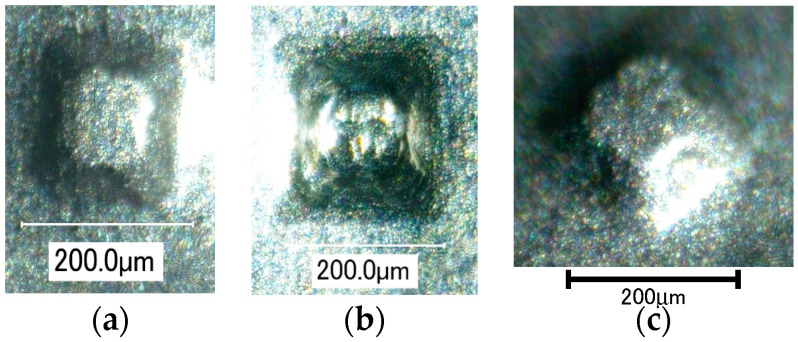
Titanium pyramids of *D* = 283 μm formed with ultrasonic vibration: (**a**) top view; (**b**) bottom view; (**c**) high angle view.

A truncated micropyramid of *D* = 283 μm formed with ultrasonic vibration is shown in Figure 16. Although the tool rotational speed *ω* was increased and table speed *v_t_* and axial feed Δ*z* were decreased for forming a smaller pyramid, its shape accuracy was not good, and partially distorted. It is seen that the radius of lateral edges was much larger than that of the forming tool and the feed marks of the tool on the back surface of the pyramid were disturbed. The size of a pyramid was reduced by a factor of five, but the foil thickness was not reduced. Hence, the bending stiffness relatively increased. This is a main reason that it was difficult to form a smaller micropyramid of titanium. 

## 4. Conclusions

Single point incremental microforming of thin shell pyramids of aluminum, stainless steel, and titanium foils was conducted under the conditions of with and without ultrasonic vibration and with and without laser heating. It was found that the laser heating can improve the forming limit of a square pyramid of 6.5 μm-thick aluminum foil and it was more effective when ultrasonic vibration was applied in addition to laser heating. The elongation obtained under the assistance of ultrasonic vibration and laser heating was 73.4%, more than 10 times as large as that obtained by tensile test. It is confirmed that the assistance of ultrasonic vibration can improve the accuracy of rather complicated shape of pyramids such as a twisted pyramid and a star pyramid. It is also found that ultrasonic vibration improves the forming accuracy of stiff materials or difficult-to-work materials even if the shapes of micropyramids are simple.

## Figures and Tables

**Table 1:** Chemical composition of 8021 aluminum foil [wt %].

Si	Fe	Cu	Mn	Mg	Zn	Others	Al
<0.15	>1.2, <1.7	<0.05	<0.05	<0.05	<0.05	<0.05	Remainder
